# The effect of HER2 expression on cisplatin-based chemotherapy in advanced non-small cell lung cancer patients

**DOI:** 10.1186/1756-9966-28-97

**Published:** 2009-07-03

**Authors:** Zuleyha Calikusu, Yesim Yildirim, Zafer Akcali, Hakan Sakalli, Nebil Bal, Ilker Unal, Ozgur Ozyilkan

**Affiliations:** 1 Department of Medical Oncology, Acibadem Maslak Hospital, İstanbul, Turkey; 2 Department of Medical Oncology, Sivas Numune Hospital, Sivas, Turkey; 3 Department of Medical Oncology, Baskent University Faculty of Medicine, Ankara, Turkey; 4 Department of Pathology, Baskent University Faculty of Medicine, Ankara, Turkey; 5 Department of Biostatistics, Cukurova University Faculty of Medicine, Adana, Turkey

## Abstract

**Introduction:**

The prognostic value of HER2 expression in patients with advanced non-small cell lung cancer remains controversial. The relationship between HER2 expression, and platinum resistance and patient survival, was investigated.

**Methods:**

Seventy-three consecutive patients (median age, 61 years) with stage IIIB and IV non-small cell lung cancer, admitted between February 2004 and December 2006, were included in this study. Sixty-one patients received gemcitabine, given as two 1250 mg/m^2 ^doses on days 1 and 8 and, cisplatin, given as a 75 mg/m^2 ^dose on day 8. Twelve patients received vinorelbine, given as two 25 mg/m^2 ^doses on day 1 and 8, and cisplatin, given as a 75 mg/m^2 ^dose on day 1. Both treatment paradigms were repeated on a 21-day cycle. Tumor response was evaluated by comparing tumor size on computerized tomography scans before and after three cycles of chemotherapy. HER2 status was examined by immunohistochemical analysis of paraffin-embedded specimens.

**Results:**

HER2 was positive in 21 of 73 patients (28.8%). Of the 21 patients with HER2 positivity, 13 (61.9%) responded to chemotherapy with either a complete response, partial remission, or evidence of stable disease. Of 52 HER2-negative patients, 48 (92.3%) exhibited a response to chemotherapy. The difference in response to therapy between HER2-positive and -negative patients was statistically significant (p = 0.003). The median overall survival duration for all patients was 13 months. Median overall survival time was 14 months for HER2-negative patients and 10 months for HER2-positive patients (log-rank p = 0.007).

**Conclusion:**

Non-small cell lung cancer patients with high expression of HER2 exhibited resistance to cisplatin-based chemotherapies that are the standard treatment for this disease. Our results indicate that HER2 status may be a predictive and prognostic factor for cisplatin- based therapy response and disease survival.

## Introduction

Non-small-cell lung cancer (NSCLC) is a leading cause of cancer deaths worldwide [1]. The prognosis of patients with advanced NSCLC remains poor despite increased understanding of the disease and therapeutic advances, heightening the need for new therapeutic approaches. Modern therapeutic strategies have achieved 1-year survival rates of up to 50% [2]. A combination of cisplatin or carboplatin with third generation agents, such as gemcitabine, paclitaxel, docetaxel, or vinorelbine, represents the standard of care for fit patients with advanced disease [3-5]. However, appreciable clinical response to chemotherapy is achieved in only 30–40% of patients, probably because of relatively higher chemoresistance intrinsic to NSCLC. The mechanism of this resistance is not well understood. Resistance does not appear to correlate with MDR1 gene expression [6], but several reports have linked NSCLC chemoresistance to mutations in TP53 and/or overexpression of HER2. The therapeutic efficacy of anticancer agents is strongly dependent on the ability of the drugs to trigger apoptosis in target tumor cells [7].

Because further advances in chemotherapy are likely to be limited, the key to improving outcomes for NSCLC patients may turn on targeted therapeutic strategies. In particular, agents that target the epidermal growth factor receptor (EGFR) may have a major impact on the treatment of advanced NSCLC [8,9].

The HER2/neu oncogene, a probable prognostic indicator in lung cancer patients, is a member of the EGFR family. Also known as c-erbB-2, HER2 is encoded by a gene located in the chromosomal region 17q11.2–q12, and encodes a transmembrane receptor-type tyrosine-protein kinase [10]. Dimerization of HER2/neu with an activated EGFR molecule activates a signal transduction cascade that leads to an increase in cell proliferation, angiogenesis, and metastatic potential, and a decrease in apoptosis. HER2/neu overexpression is found more often in breast, ovarian, and lung cancer, especially adenocarcinoma [10], and can be detected by immunohistochemistry (IHC). Clinical trials indicate that angiogenesis is more active in tumor tissues in which HER2 is activated, and have suggested that this may lead to platinum resistance [11,12]. Tsai and colleagues, using a panel of 20 NSCLC lines obtained from untreated patients, found that overexpression of HER2 was a marker for intrinsic multidrug resistance [6]. HER2-mediated chemoresistance depended on the type of drug used, cell type, and HER2 expression level [10]. The aim of the current study was to investigate the relationship between HER2 expression in non-small cell lung cancer patients, and to assess the effect of this expression on cisplatin-based chemoresistance.

## Patients and methods

### Patients

Seventy-three consecutive, previously untreated advanced non-small cell lung cancer patients referred to Baskent University Medical Faculty Medical Oncology Department between February 2004 and December 2006 were included in the study. All patients were diagnosed with stage IIIB with pleural effusion or stage IV, according to the American Joint Committee on Cancer staging system (AJCC) 1997. The performance status of patients was 0–2 according to the Eastern Cooperative Oncology Group (ECOG) scale. The studied patients included four females and 69 males with a median age of 61 years (range, 35–78 years). Bone marrow, renal and hepatic functions were sufficient for patients to take part in the study. Two-dimensional lesions, measurable by radiologic imaging and physical examination, were taken into account for follow-up criteria. Patients with no measurable masses and concomitant life-threatening diseases were not included in the study.

### Treatment

Sixty-one patients received gemcitabine, given as two 1250-mg/m^2 ^doses on days 1 and 8 and, cisplatin, given as a 75-mg/m^2 ^dose on day 8 [13]. Twelve patients received vinorelbine given as two 25-mg/m^2 ^doses on day 1 and 8 and, cisplatin, given as a 75-mg/m^2 ^dose on day 1. Both gemcitabine/cisplatin and vinorelbine/cisplatin treatment paradigms were repeated on a 21-day cycle. Patients received a total of four to six chemotherapy courses. Twenty patients received palliative radiotherapy; eight received radiotherapy for bone metastases and twelve received radiotherapy for cranial metastases before the first chemotherapy course.

### Treatment evaluation

Prior to treatment, all patients were evaluated by physical examination, electrocardiography, chest X-ray, bone scintigraphy, thorax computerized tomography (CT), and upper abdominal ultrasound and CT; complete blood counts were also performed. Cranial computerized tomography or magnetic resonance imaging was performed in patients with signs or symptoms of central nervous system disease.

Tumor response was evaluated after the third chemotherapy course by comparison of tumor size on CT scans before and after chemotherapy. We used World Health Organization (WHO) guidelines for response criteria throughout the study. Objective response was defined as follows: complete response (CR) – complete resolution of all disease lasting at least 1 month; partial response (PR) – a decrease ≥ 50% lasting at least 1 month; stable disease (SD) – a decrease of < 50% or an increase of < 25% in lesions; and progressive disease (PD) – 25% or more increase in the size of one or more lesions, or the appearance of new lesions.

### IHC

Tumor-containing tissue slices for examination by IHC were selected from archived paraffin-embedded pathology laboratory specimens. Five-micron thick slices were deparaffinized, and then processed for antigenic retrieval by suspending in a 10-mM citrate buffer solution (pH 6.0) and boiling in a microwave oven for 5 minutes at 500 W, 5 minutes at 400 W and 5 minutes at 350 W. Specimens were kept in a 3% hydrogen peroxide solution to remove endogenous peroxides, and then incubated for 5 minutes with Ultra V block (TP-125-HU, Thermo Fisher Scientific Inc., USA) to reduce background. A solution of HER2 antibody (Clone e2-4001 + 3B5, Ready to Use for Immunohistochemical Staining, NeoMarkers/Labvision, USA) was added drop-wise to the slices and incubated for 45 minutes at room temperature. After washing for 10 with Tris-buffered saline (TBS), biotin-conjugated TP-125-HB (goat anti-polyvalent) was applie and allowed to stand for 10 minutes. Slide- mounted slices were again washed with TBS (10 minutes) and then incubated with streptavidin peroxide for 15 minutes. Slices were then washed for 10 minutes with TBS, and 3-amino-9-ethylcarbazole (AEC) chromogenic substrate (RTU lot: 065020) was added dropwise. Slices were stored in the dark after counterstaining with Mayer's Hematoxylin. Under a light microscope, brown-red coloration in tumor cytoplasmic membranes was considered HER2 positive. Unstained membranes were considered negative (-); pale and partial membranous staining in less than 10% of tumor cells was given a score of 1+; pale and complete staining in more than 10% of tumor cells was given a score of 2+; and strong and complete staining in more than 10% of tumor cells was given a score of 3+.

### Statistical analysis

SPSS (Statistical Package for Social Sciences) version 16 was used to analyze the results. After descriptive statistical analyses, survival curves were drawn according to the Kaplan Meier method. The differences between survival curves were analyzed using log-rank tests. Chi-square tests were used to investigate differences between proportions. The effects of histopathology, HER2-positivity and stage of disease on survival were investigated using a Cox Regression Model. Values of p < 0.05 were considered statistically significant.

## Results

### Patient characteristics

Seventy-three patients with non-small cell lung cancer were evaluated between February 2004 and December 2006. Thirty patients (41%) had stage IIIB disease, and 43 (59%) stage IV. Histopathological types were squamous cell carcinoma in 34 patients (46.5%), adenocarcinoma in 27 (37%) and histopathologically non-small cell lung cancer not otherwise specified (NOS) in 12 (16.5%).

The median follow-up time was 13 months (range, 2–44 months). At the end of follow-up, 66 patients (90.4%) had died and 7 (9.6%) survived. During the follow-up period, metastases were detected in bone (13 patients), brain (10 patients), adrenal gland (2 patients), pericardium (1 patient), and leptomeninges (1 patient).

### HER2 expression and response to chemotherapy

Tumors were HER2-positive in 21 of 73 patients (28.8%); of these, 5 patient specimens were scored as 1+, 10 2+ and 6 3+. IHC staining for HER2 in relation to clinical characteristics of patients and histological tumor type is shown in Table 1. There was no correlation between the expression of HER2 and the age of patients, stage of tumor, or histological tumor type. One patient showed a complete response (CR) to chemotherapy, and 32 patients exhibited partial response (PR). Disease stabilization (SD) was confirmed in 28 patients, and progressive disease (PD) was manifest in 12. For purposes of statistical analysis, CR, PR, and SD were evaluated together as a single group and PD was evaluated separately as a second group. Of the HER2-positive patients, 61.9% (13/21) showed a response to chemotherapy (CR+PR+SD); among HER2-negative patients, 92.3% (48/52) responded to chemotherapy. The response to therapy was significantly lower in HER2-positive patients than in HER2-negative patients (p = 0.003, chi-squared test; Table 2). There was no correlation between the response to chemotherapy and clinical characteristics of patients, stage of tumor, or histological type (Table 3).

**Table 1:** Immunohistochemical staining for HER 2 according to clinical characteristics of patients, stage and histological type of tumor

Patient characteristics	Number of patients	HER 2 +(%)
Total Patients	73	21 (28.8)

Sex		
Male	69	19 (27.5)
Female	4	2 (50)

Stage		
Stage IIIB	30	9 (30)
Stage IV	43	12 (27.9)

Histopathology		
Adenocarcinoma	27	11 (40.7)
Squamous cell (Epidermoid)	34	5 (14.7)
Not otherwise specified (NOS)	12	5 (41.6)

**Table 2:** Response to chemoterapy according to expression of HER 2

HER 2	CR+PR+SD	PD
HER 2 (+)	13 (63.9)	8(38.1%)

HER 2 (-)	48 (92.3%)	4(7.7%)

**Table 3:** Response to chemoterapy according to clinical characteristics of patients and histological type of tumor

Patient characteristics	Number of patients	CR+PR+SD	PD
Total Patients	73	61(83.6%)	12 (16.4%)

Sex			
Male	69	58 (84%)	11 (16%)
Female	4	3(75%)	1 (25%)

Stage			
Stage IIIB	30	29(96.6%)	1(3.4%)
Stage IV	43	32 (74.4%)	11 (25.6%)

Histopathology			
Adenocarcinoma	27	21(78%)	6(22%)
Squamous cell (Epidermoid)	34	31(91.2%)	3 (8.8%)
Not otherwise specified (NOS)	12	9 (75%)	3 (25%)

### Survival

**Figure 1:**
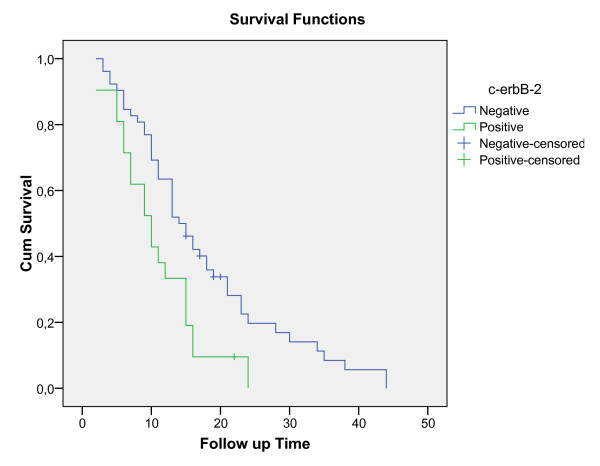
**Overall survival for the c-erbB-2 (-) and c-erbB-2 (+) patients (months), Kaplan-Meier curve**.

Median overall survival for all 73 patients was 13 months. For Her2-negative patients, median overall survival was 14 months, whereas for HER2-positive patients, median overall survival was 10 months, a difference that was statistically significant (p = 0.007, log-rank test). Survival curves are shown in Figure 1. One-year survival probabilities were 76.9% for HER2-negative patients and 42.9% for HER2-positive patients; the corresponding 2-year survival rates were 51.9% and 0%, respectively.

### Cox's regression analyses

After correcting for age, gender, and stage, HER2 positivity was found to increase the individual death risk by 2.104-fold (95% CI: 1.206–3.670; p = 0.009).

## Discussion

In this study, we detected HER2 overexpression in 22 of 73 tumors (28.8%) using immunohistochemistry. The mean percentage of non-small cell lung carcinomas reported to overexpress HER2 ranges from 18–55%, with an average of 31% [14]. This diversity of results probably reflects differences in methodologies, which have included flow cytometry, IHC, and Western blotting. Moreover, the cut-off point for HER2 positivity varied among studies, ranging from 5% to 10% [15,16]. In our study, we used 10% as the cut-off point. Patients with a HER2 positivity score of +1 to +3 by IHC staining criteria were defined as HER2-positive. The frequency of HER2 staining differed among non-small cell lung cancer subtypes, and was much higher for adenocarcinoma than for squamous or large-cell carcinomas [14-17]. We observed similar results in our study.

Trastuzumab, a monoclonal antibody that binds to HER2, was originally developed for use against breast cancer. Recently, a number of phase II trials have been conducted to evaluate the response of NSCLC to trastuzumab [18]. Some of these trials enrolled lung cancer patients with +2 or +3 HER2 expression scores; however, others included patients with tumor HER2-positive scores of +1 to +3 [18]. Because of these differences in enrollment criteria, it is not clear to what degree HER2 overexpression is a prerequisite for trastuzumab effectiveness.

There have been conflicting reports on the prognostic value of HER2 overexpression. Recently, Nakamura and colleagues published a meta-analysis to assess the association of HER2 overexpression with prognosis in NSCLC [19]. A total of 2,579 patients were included in the final analysis, which concluded that survival at 3 and 5 years was significantly poorer in patients with HER2 overexpression [19].

Different hypotheses have been proposed to explain the poor prognosis of patients with HER2-overexpressing tumor cells. One suggestion is the intrinsic resistance to cytotoxic agents is high in HER2-expressing tumor cells. It is known that high levels of HER2 expression in breast cancer predict resistance to adjuvant chemotherapy [20], and HER2 overexpression has been associated with poor prognosis in breast cancer [21]. The intrinsic chemoresistance of HER2-overexpressing NSCLC lines was investigated by Tsai and associates, who showed that resistance to the cytotoxicity of doxorubicin and cisplatin increased with greater expression of HER2 [6].

Recently, investigations into the relationship between p53 and HER2 expression, and response to neoadjuvant chemotherapy, in resected lung cancer demonstrated a tendency toward chemoresistance in tumors with a high level of HER2 expression [16]. Although cisplatin-based combination chemotherapies are the standard treatment for NSCLC [3], our study clearly showed a lower response to cisplatin-based chemotherapy in HER2-positive patients than in HER2-negative patients. The median overall survival was also reduced in HER2-positive patients. These results suggest that NSCLC patients with HER2-overexpressing tumors may require a more potent chemotherapy regimen to achieve longer survival. HER2 status thus seems to be both a predictive and a prognostic factor for cisplatin- based therapy response and disease survival.

Immunohistochemistry is a commonly used method to detect HER2 in different tumor types. Fluorescence *in situ *hybridization (FISH), another method often used to evaluate HER2 status, mainly determines HER2 gene copy number [22]. Recently, comparisons of IHC and FISH techniques in breast cancer have shown that FISH is more specific than IHC [22]. In NSCLC, the optimal technique for showing HER2 overexpression has not yet been determined. Unlike the situation in breast cancer, HER2 overexpression in NSCLC is more likely caused by chromosomal duplication rather than gene amplification [23]. Recently, Kuyama and co-workers investigated the relationship between HER2 expression and treatment outcome in locally advanced lung carcinoma using both methodologies [24]. The HER2-FISH results were marginally correlated with IHC results, and only the HER2-FISH data were determined to be an independent factor for poor prognosis of cisplatin-based chemotherapy and survival [24]. In our study, we measured HER2 protein expression by IHC. Although FISH results are demonstrably better for determining HER2 status in breast cancer, until it becomes clear which method is better for evaluating HER2 status in NSCLC, IHC remains a widely available, simple, and less expensive method for determining HER2 expression.

## Conclusion

Despite advances in chemotherapy, the prognosis for NSCLC patients remains poor. Many factors, including HER2 overexpression, may contribute to this adverse outcome Only a few studies have correlated HER2 status and cisplatin-based chemotherapy resistance. Here, we showed that advanced NSCLC that express a high level of HER2 are resistant to cisplatin-based chemotherapies, which are the standard for this disease. HER2 status thus appears to represent both a predictive and prognostic factor for advanced NSCLC.

## Competing interests

The authors declare that they have no competing interests.

## Authors' contributions

ZC participated in coordination of the study. YY participated in the design of the study and drafted the manuscript. ZA participated in the sequence alignment. HS paricipated in the sequence alignment. NB participated in the pathological examination. IU performed the statistical analysis. OO participated in its design and coordination.
